# The association between ABO blood types and peripherally inserted central catheter-related venous thrombosis for patients with cancer: A retrospective 7-year single-center experience and meta-analysis

**DOI:** 10.1371/journal.pone.0305746

**Published:** 2024-07-01

**Authors:** Xiao-Hong Wu, Yu Xiao, Ren-Di Tian

**Affiliations:** 1 Nursing Department, Sichuan Cancer Hospital & Institute, Sichuan Cancer Center Affiliate Cancer Hospital of University of Electronic Science and Technology of China, Chengdu, China; 2 Psychosomatic Medical Center, The Clinical Hospital of Chengdu Brain Science Institute, MOE Key Lab for Neuroinformation, University of Electronic Science and Technology of China, Chengdu, China; 3 Psychosomatic Medical Center,The Fourth People’s Hospital of Chengdu, Chengdu, China; Stead Family Children’s Hospital, Carver College of Medicine, University of Iowa, UNITED STATES

## Abstract

**Background:**

This meta-analysis evaluated the association of ABO blood type on central venous catheter-related thrombosis (CRT).

**Methods:**

Data were derived from 8477 patients at Sichuan Cancer Hospital from January 2015 to December 2021 and articles previously published in Chinese and English databases. Data from our hospital were collected by reviewing electronic medical records. Searched databases included CNKI, VIP, Wan Fang, China Biomedical, PubMed, Cochrane Library, Web of Science, EMBASE, CINAHL, and OVID (up to July 2023). All statistical analyses were performed using SPSS 22.0 and Revman 5.3. The Bonferroni method was used to adjust the α test level for reducing the risk of I errors in the multiple comparisons. A P-value < 0.05 was considered statistically significant. Continuous variables were analyzed using a two-independent sample T test. The chi-squared test was used to analyze categorical data.

**Results:**

A total of 818 studies were identified in the search. However, only four studies met the inclusion criteria. Combined with data from our hospital, five studies were included with a total of 18407 cases. Those studies only focused on peripherally inserted central catheter (PICC). According to the data from our hospital, logistic regression revealed that myelosuppression [odds ratio (OR), 1.473; P = 0.005) and radiotherapy(OR, 1.524; P<0.001) were independent risk factors for symptomatic PICC- VTE. Blood types A (OR, 1.404; P = 0.008), B (OR, 1.393; P = 0.016), and AB (OR, 1.861; P<0.001) were associated with a significantly higher risk of symptomatic PICC-VTE than blood type O. And the hematologic tumor has a significantly higher risk of PICC-VTE than breast cancer (OR, 0.149; P < 0.001), and gynecological tumor (OR, 0.386; P = 0.002). In the meta-analysis of the association between ABO blood type and PICC related thrombosis, the I2 statistic was not significant in any of the pairwise comparisons, and a fixed-effects model was subsequently used for all analyses. The meta-analysis indicated that the incidence of symptomatic PICC related thrombosis was significantly lower in individuals with the O blood type (3.30%) than in those with the A (4.92%), B (5.20%), or AB (6.58%) blood types (all P < 0.0083). However, in the pairwise comparisons among A, B, and AB, the differences were nonsignificant (P > 0.0083).

**Conclusions:**

According to the results from our single center analysis, we found that myelosuppression, radiotherapy, hematologic tumor, and non-O blood type were independent risk factors for symptomatic PICC related thrombosis. In the meta-analysis of further exploration of ABO blood type and PICC related thrombosis, we found that ABO blood type may influence PICC related thrombosis, and individuals with the O blood type had a lower risk of PICC related thrombosis than those with non-O blood type.

## Introduction

A central venous catheter (CVC) is the predominant infusion device used for patients with cancer undergoing chemotherapy, effectively reducing the risk of drug extravasation and peripheral vascular stimulation. There are four types of CVCs: non-tunneled CVCs, tunneled CVCs, peripherally inserted central catheter (PICC), and totally implanted venous access port (TIVAP). A hypercoagulable state and chemotherapy-induced damage to the vascular endothelium increase the risk of catheter-related thrombosis (CRT) in these patients[1,2]. CRT can also cause pulmonary embolisms[3,4]. Moreover, CRT is one of the most common complications of CVCs and can lead to catheter occlusion. The incidence of catheter-associated thrombosis is 2%-22%[5,6]. Hence, the prevention and treatment of CRT in patients with cancer have been a major focus of research.

Common risk factors for CRT include damage to the vascular wall during catheterization, previous history of VTE, D-dimer levels, and catheter-to-vein ratio[7,8]. However, few studies have assessed the association between the ABO blood type and CRT, and the results remain controversial[9–13]. One study found no association between the ABO blood type and CRT[13], whereas another study found that, compared with the O blood type, only the B blood type increased the incidence of CRT[9]. Moreover, these studies only compared the incidence of CRT between the O and non-O blood types without conducting pairwise comparisons. Therefore, we do not know whether differences are present in the incidence of CRT between blood types A, B, and AB. This meta-analysis aimed to systematically evaluate whether ABO blood type influence CRT by using existing studies and a large sample from our hospital, and conducting pairwise comparisons to determine which blood type has the lowest incidence of CRT. This will provide a reference for the future prevention and treatment of CRT.

## Methods

This review was conducted according to the PRISMA extension statement and has been already registered on the PROSPERO database (CRD42022312956)[14].

### Data from our hospital

A retrospective analysis was conducted on the clinical data of patients with malignant tumors who underwent PICC placement in the venous catheter outpatient department of Sichuan Cancer Hospital from January 2015 to December 2021. All data for the entire catheterization period were collected by reviewing electronic medical records. The following data were collected: age, sex, insertion arm, pain, chronic obstructive pulmonary disease (COPD), myelosuppression, hyperlipidemia, hypertension, diabetes, radiotherapy, cancer diagnosis, activated partial thromboplastin time (APTT), and ABO blood type.

**Fig 1:**
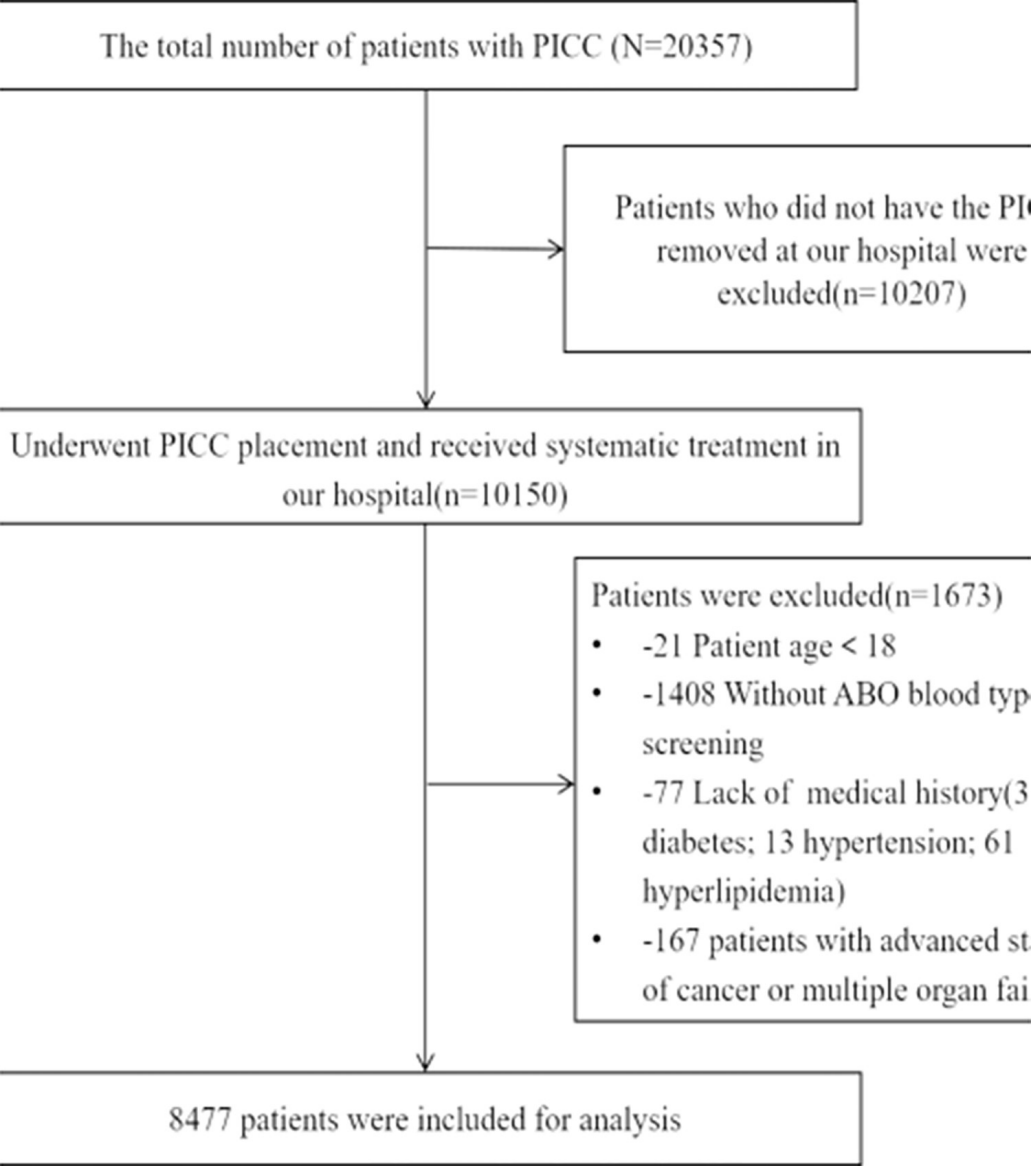
The flow diagram of patients selection.

Inclusion criteria: age≥18 years; underwent PICC placement and received systematic treatment in our hospital; ABO blood type screening conducted in our hospital. Exclusion criteria: Patients who did not have the PICC removed at our hospital were excluded because their data for the entire period of catheterization could not be collected by follow-up from the electronic medical record system; patients with advanced stages of cancer, and patients with multiple organ failure were excluded because their blood was in an extremely hypercoagulable state. Finally, 8477 patients were included. The flow diagram of patients selection was shown in Fig 1. The Sichuan Cancer Hospital Ethics Committee approved data acquisition (No. SCCHEC-02-2023-115).

In this study, all patients with PICC related thrombosis were all symptomatic thrombosis. Throughout the catheterization period, color Doppler ultrasonography was performed for patients with clinical symptoms of suspected PICC related thrombosis: arm swelling, skin color change, arm pain, increased skin temperature, or arm numbness on the side of the catheter. Criteria for ultrasound diagnosis of PICC related thrombosis were: (1) intravascular solid echo; (2) insufficient or complete absence of lumen blood flow signal; and (3) venous incompressibility or partial closure after compression. Meeting any of the above criteria is considered as PICC related thrombosis[15].

All the patients underwent catheter placement in the venous catheter outpatient department. All catheter placement nurses had experience with >500 cases. Catheter placement was performed under B-mode ultrasound guidance combined with the Seldinger technique. A Bard Groshong PICC or Power PICC was used for all catheter placements. The basilic vein was the preferred site for catheter placement, followed by the brachial and cephalic veins. During catheter placement, ultrasound examination of the internal jugular and subclavian veins was routinely performed to determine the catheter trajectory. After the catheter placement, a 5×8 hydrocolloid dressing was applied above the puncture site to prevent catheter-related venous inflammation. After the procedure, the tip position was confirmed by radiography, and immediate catheter adjustment was performed for any catheter misplacement. Catheter maintenance was performed every seven days after the procedure, and all patients underwent routine ultrasound examination before PICC removal. A nurse qualified for catheter maintenance performed the PICC removal in a sterile state. A sterile transparent dressing was applied to cover the puncture site for 24h after PICC removal.

### Search strategy

Clinical questions were presented using the PICO framework, as shown in Table 1. The Chinese databases searched included the CNKI, VIP, Wan Fang, and China Biomedical databases. The foreign databases included PubMed, the Cochrane Library, Web of Science, EMBASE, the Cumulative Index to Nursing and Allied Health Literature (CINAHL), and OVID (up to 13 July, 2023). The search strategy involved using free text terms and Medical Subject Headings (Mesh), together with boolean operators. The PubMed database was used as an example for a detailed search strategy (Table 2). Additionally, the references of related articles were examined to identify additional articles that met the inclusion criteria.

**Table 1:** PICO framework.

Population	Patients with central venous catheter
Intervention	Different ABO blood type
Comparison	Different ABO blood type
Outcome	Central venous catheter related vein thrombosis

**Table 2:** Example of PubMed search strategy.

ID	Search Strategy	Result
#1	(((((((((((((((((((((ABO Blood-Group System[MeSH Terms]) OR (ABO Blood-Group System[Title/Abstract])) OR (ABO Blood Group System[Title/Abstract])) OR (ABO Blood-Group Systems[Title/Abstract])) OR (Blood-Group System, ABO[Title/Abstract])) OR (Blood-Group Systems, ABO[Title/Abstract])) OR (System, ABO Blood-Group[Title/Abstract])) OR (Systems, ABO Blood-Group[Title/Abstract])) OR (H Blood Group System[Title/Abstract])) OR (H Blood Group[Title/Abstract])) OR (Blood Group, H[Title/Abstract])) OR (Blood Groups, H[Title/Abstract])) OR (H Blood Groups[Title/Abstract])) OR (ABH Blood Group[Title/Abstract])) OR (ABH Blood Groups[Title/Abstract])) OR (Blood Group, ABH[Title/Abstract])) OR (Blood Groups, ABH[Title/Abstract])) OR (Blood Group H Type 1 Antigen[Title/Abstract])) OR (ABO Factors[Title/Abstract])) OR (ABO Factor[Title/Abstract])) OR (Factor, ABO[Title/Abstract])) OR (Factors, ABO[Title/Abstract])	16419
#2	(((((((((((((((((((((((((((central venous catheters[MeSH Terms]) OR (Catheter, Central Venous[Title/Abstract])) OR (Catheters, Central Venous[Title/Abstract])) OR (Venous Catheter, Central[Title/Abstract])) OR (Venous Catheters, Central[Title/Abstract])) OR (Central Venous Catheter[Title/Abstract])) OR (port[Title/Abstract])) OR (totally implanted venous infusion port[Title/Abstract])) OR (totally implantable venous access port[Title/Abstract])) OR (totally implantable catheter[Title/Abstract])) OR (totally implanted venous access device[Title/Abstract])) OR (fully implantable catheter[Title/Abstract])) OR (totally implantable long-term central vascular access device[Title/Abstract])) OR (implantable venous access port[Title/Abstract])) OR (totally implantable venous device[Title/Abstract])) OR (venous access port[Title/Abstract])) OR (inserted central venous catheter[Title/Abstract])) OR (port a cath[Title/Abstract])) OR (implanted port[Title/Abstract])) OR (infusion port[Title/Abstract])) OR (peripherally implanted central venous catheter[Title/Abstract])) OR (peripherally inserted central catheter[Title/Abstract])) OR (TIVAP[Title/Abstract])) OR (TIVAD[Title/Abstract])) OR (TIAP[Title/Abstract])) OR (CVC[Title/Abstract])) OR (PICC[Title/Abstract])) OR (CAVD[Title/Abstract])	46012
#3	(((((((((((thrombosis[MeSH Terms]) OR (thrombosis[Title/Abstract])) OR (thromboses [Title/Abstract])) OR (thrombi[Title/Abstract])) OR (thrombus[Title/Abstract])) OR (blood clot[Title/Abstract])) OR (blood clots[Title/Abstract])) OR (thromboembolism [Title/Abstract])) OR (venous thromboembolism[Title/Abstract])) OR (deep vein thrombosis[Title/Abstract])) OR (VTE[Title/Abstract])) OR (CRT[Title/Abstract])	320655
#4	#1 AND #2 AND #3	4

### Inclusion and exclusion criteria

All randomized controlled trials (RCTs), cohort studies, and case-control studies on the relationship between the ABO blood type and CVC-related VTE in patients with cancer from both English and Chinese literature were included. Abstracts, unavailable full texts, unpublished articles, letters, reviews, republished articles, animal studies, biologic studies, and studies with unclear methodologies or outcomes were excluded.

### Study selection

**Fig 2:**
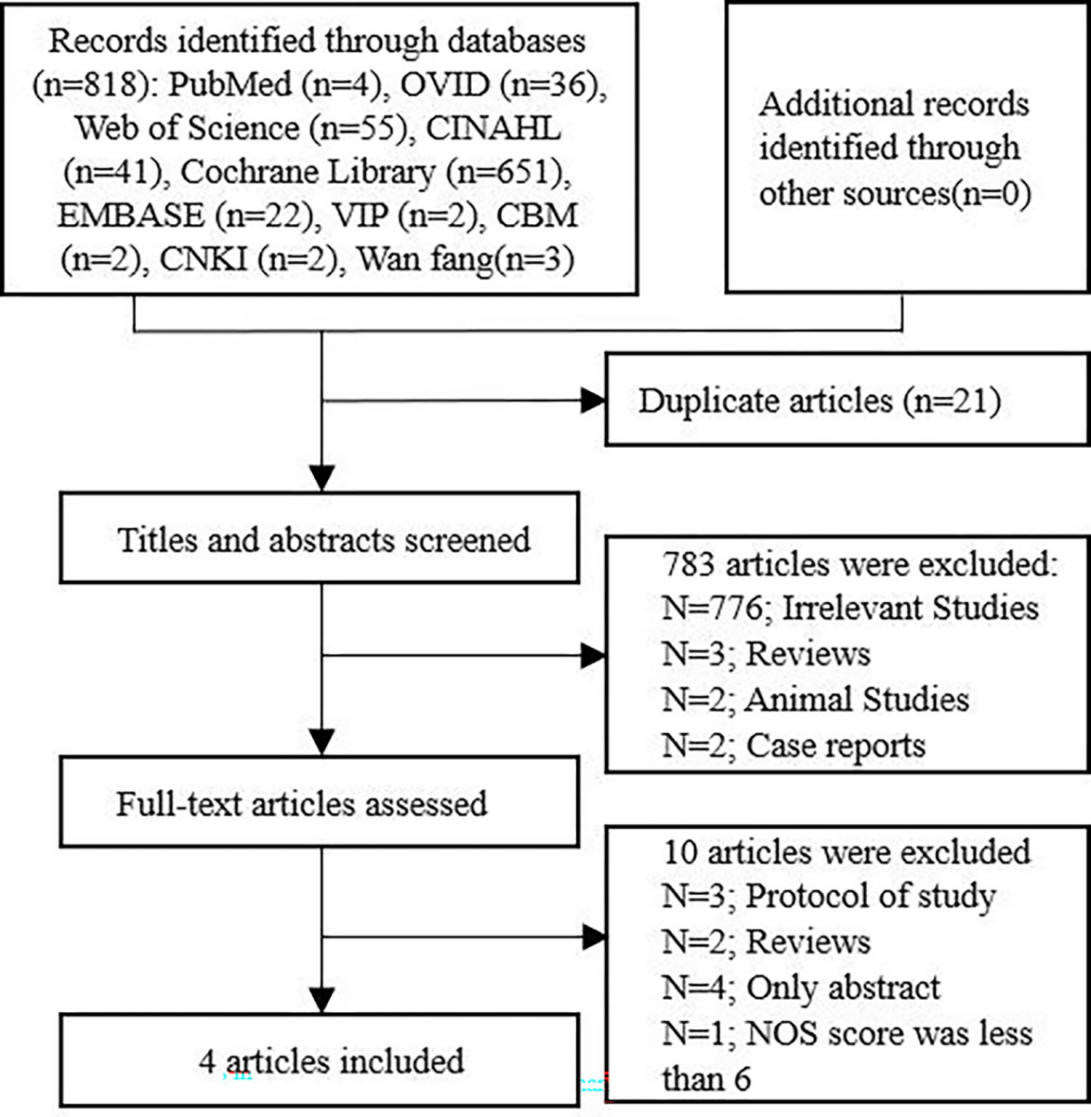
Flow diagram of study selection.

All retrieved literature was managed using EndNote software. The literature selection was performed independently by two reviewers (WXH and XY). EndNote was used to select duplicate articles. The titles and abstracts of all articles were screened by two reviewers (WXH and XY) to identify related studies. The full-text reports of the related studies were evaluated by the two reviewers to identify those that met the inclusion criteria (WXH and XY). The selection process is illustrated in Fig 2. Any disagreements were resolved through discussions between the reviewers or consultation with a third reviewer (TRD).

### Outcomes

The only outcome indicator included in this meta-analysis was the central-venous catheter-related thrombosis. Catheter-associated thrombosis is defined as the formation of blood clots in the outer wall of the catheter and the inner wall of the blood vessel, including symptomatic and asymptomatic CRT[10].

### Quality assessment

The quality of the articles was independently assessed by two reviewers (WXH and XY). The risk of bias in the cohort and case-control studies was evaluated using the Newcastle-Ottawa Scale (NOS), which comprises eight items with a total score of nine points[16]. A score of more than six was classified as level A, whereas a score of less than six was considered level B. Randomized controlled trials (RCTs) were conducted using the criteria outlined in the Cochrane Handbook[17]. The handbook contains seven items, and each item is rated as low, high, or unclear. Low risk represented high quality. The ratings for the RCTs were A (all items rated as low risk), B (some items rated as low risk), and C (all items rated as high risk). Studies with a rating of C or a score of < 6 points were excluded.

### Data abstraction

Data were extracted by two reviewers (WXH and XY) who read the full texts separately. Any disagreements during data extraction were resolved through discussion between the reviewers (WXH and XY) or consultation with a third reviewer (TRD). The following data were extracted from the included studies: name(s) of the author(s), year of publication, country, study design, type of catheter, clinical setting, study period, number of thrombosis cases, VTE type, ABO blood type, and other relevant data. If the necessary data were incomplete or missing, our team contacted the corresponding author to obtain the missing information. If two identical studies were published from the same institution, the study with the longest follow-up and largest sample size was included in the review

### Data analysis

Data analysis was conducted using SPSS (version 22.0; IBM, Armonk, NY, USA) and Review Manager version 5.3. Descriptive statistics were used to summarize the characteristics of the patients included in this study. In part of analysis for our single center data, continuous variables with a normal distribution (means ± standard error) were analyzed using a two-independent sample T test. The chi-squared test was used to analyze categorical data. Binary logistic regression was used to estimate the risk factors associated with PICC–VTE. Statistically significant indicators from the univariate analysis were included in binary logistic regression to analyse. The odds ratio (OR) was used for effect analysis, with each effect dose providing a 95% confidence interval (CI). In the meta-analysis, heterogeneity among included studies was tested by X2. I2 represents heterogeneity. If the I2 statistic showed no statistical heterogeneity among the results, a fixed-effects model was used for meta-analysis. Otherwise, a random-effects model was used. In the multiple comparisons, the Bonferroni method was used to adjust the α test level (correcting α = 0.05/the number of pairwise comparisons), for reducing the risk of I errors, and P < 0.0083 was statistically significant. In the non-pairwise comparison analysis, a P-value < 0.05 was considered statistically significant. A funnel plot was used to assess publication bias. A symmetrical and even distribution of dots in the funnel on both sides of the vertical line representing X = 0 indicated a low possibility of an obvious small-sample effect or publication bias.

## Results

### The analysis of risk factors for PICC-VTE in our hospital

**The general characteristics of patients.** A total of 8477 patients met the inclusion criteria in our hospital from January 2015 to December 2021. The patient characteristics are shown in Table 3. The incidence of symptomatic PICC-VTE was 5.01% (425/8477). Male accounted for 57.61% (4884/8477). In addition, right-sided catheter placement accounted for 61.35% (5201/8477). The largest proportion of cases (36.86%) were in patients with the digestive system tumors. In terms of blood type, the largest proportion (33.81%) was observed in patients with the blood type O. The second largest proportion (32.82%) was observed in patients with blood type A. (Table 3)

**Table 3:** Univariate analysis for PICC-VTE.

	PICC related thrombosis		Total (n = 8477)	X2/T	P
	NO(n = 8052)	YES(n = 425)			
**Age(years)**	55.87±11.20	55.96±11.49		0.163	0.87*
Sex					
Male	4593(94.04%)	291(5.96%)	4884	21.593	<0.001**
Female	3459(96.27%)	134(3.73%)	3593		
Insertion arm					
Left	3104(94.75%)	172(5.25%)	3276	0.628	0.428**
Right	4948(95.14%)	253(4.86%)	5201		
Pain					
NO	5551(95.36%)	270(4.64%)	5821	5.492	0.019**
YES	2501(94.16%)	155(5.84%)	2656		
**COPD**					
NO	7855(94.92%)	420(5.08%)	8275	2.8	0.094**
YES	197(97.50%)	5(2.50%)	202		
**Myelosuppression**					
NO	2423(96.27%)	94(3.73%)	2517	12.297	<0.001**
YES	5629(94.45%)	331(5.55%)	5960		
**Hyperlipidemia**					
NO	7799(95.01%)	410(4.99%)	8209	0.198	0.656**
YES	253(94.40%)	15(5.60%)	268		
**Hypertension**					
NO	6829(94.87%)	369(5.13%)	7198	1.276	0.259**
YES	1223(95.62%)	56(4.38%)	1279		
**Diabetes**					
NO	7493(94.90%)	403(5.10%)	7896	1.972	0.16**
YES	559(96.21%)	22(3.79%)	581		
**Radiotherapy**					
NO	4957(96.27%)	192(3.73%)	5149	45.454	<0.001**
YES	3095(93.00%)	233(7.00%)	3328		
**Cancer diagnosis**					
Breast cancer	986(98.70%)	13(1.30%)	999	63.941	<0.001***
Digestive system neoplasms	2979(95.33%)	146(4.67%)	3125		
Lung cancer	1038(93.94%)	67(6.06%)	1105		
Hematologic tumor	191(92.27%)	16(7.73%)	207		
Head and neck cancer	1351(92.09%)	116(7.91%)	1467		
Gynecological tumor	1240(95.98%)	52(4.02%)	1292		
Other cancer	267(94.68%)	15(5.32%)	282		
**APTT**	28.21±6.86	27.79±6.27		1.34	0.181*
Blood type					
O	2755(96.13%)	111(3.87%)	2866	15.367	0.002***
A	2632(94.61%)	150(5.39%)	2782		
B	1974(94.63%)	112(5.37%)	2086		
AB	691(93%)	52(7%)	743		

COPD: Chronic obstructive pulmonary disease

APTT Activated partial thromboplastintime

*:Two independent samples T test

**:2 × 2 Chi-square test

***:2 × C Chi-square test

#### The univariate analysis of risk factors for PICC-VTE

The incidence of PICC related thrombosis was significantly higher in male patients than that in female patients(3.73% vs. 5.96%, P < 0.001), in patients with pain than that in patients without pain (4.64% vs. 5.84%, P = 0.019), in patients with myelosuppression than that in patients without myelosuppression (3.73% vs. 5.55%, P<0.001), and in patients undergoing radiation therapy than that in patients without radiation therapy (3.73% vs. 7.00%, P<0.001). The incidence of PICC-VTE, cancer diagnosis, and the ABO blood type (all P<0.05) were significantly correlated. (Table 3)

#### The multivariate analysis of risk factors for PICC-VTE

Multivariate logistic regression revealed myelosuppression(OR, 1.473; 95% CI, 1.124–1.931; P = 0.005) and radiotherapy (OR, 1.524; CI, 1.206–1.925; P<0.001) were found to be independently risk factors of PICC-VTE. The A (OR, 1.404; 95% CI, 1.091–1.808; P = 0.008), B (OR, 1.393; CI, 1.063–1.826; P = 0.016), and AB (OR, 1.861; CI, 1.322–2.621; P<0.001) blood types has a significantly higher risk of PICC-VTE than O blood type. And the hematologic tumor has a significantly higher risk of PICC-VTE than breast cancer (OR, 0.149; 95% CI, 0.07–0.315; P < 0.001), and gynecological tumor (OR, 0.386; 95% CI, 0.213–0.701; P = 0.002). (Table 4)

**Table 4:** Multivariate logistic analyses for PICC-VTE.

	OR (95% CI)	P
**Myelosuppression**	1.473(1.124–1.931)	0.005
**Radiotherapy**	1.524(1.206–1.925)	<0.001
**Blood type**		
O	Reference	0.002
A	1.404(1.091–1.808)	0.008
B	1.393(1.063–1.826)	0.016
AB	1.861(1.322–2.621)	<0.001
**Cancer diagnosis**		
Hematologic tumor	Reference	<0.001
Digestive system neoplasms	0.614(0.355–1.064)	0.082
Lung cancer	0.741(0.416–1.319)	0.307
Breast cancer	0.149(0.07–0.315)	<0.001
Head and neck cancer	0.763(0.432–1.345)	0.349
Gynecological tumor	0.386(0.213–0.701)	0.002
Other cancer	0.614(0.293–1.283)	0.194

OR: Odds Ratio

### Systematic review

#### Search process and quality assessment

A total of 818 studies were initially identified from 10 databases. Studies were excluded according to specific criteria, and the detailed reasons for exclusion are shown in Fig 2. Five relevant studies were selected for this review. However, one study was excluded because this had a NOS score of < 6, indicating lower quality. Combined with the present study from our hospital and the four retrieved studies from Chinese and English databases, five studies with 18407 cases met the inclusion criteria and were included in this review, all of which were rated as level A (Table 5).

**Table 5:** Risk of bias for non-RCT studies based on the Newcastle-Ottawa scale.

Study	The selection of study sample				Comparability	Exposure factors		Quality score	Level	Whether or not to retain
	①	②	③	④	⑤	⑥	⑦			
Our study(2023)	1	1	1	1	1	2	1	8	A	YES
Haddad et al.(2018)	1	0	0	1	1	1	1	5	B	NO
Koo et al.(2018)	1	1	1	1	1	2	1	8	A	YES
Li et al.(2021)	1	1	1	1	1	2	1	8	A	YES
Wang-1 et al.(2019)	1	1	1	1	1	2	1	8	A	YES
Wang-2 et al.(2019)	1	1	1	1	1	2	1	8	A	YES

①Definition of Case; ②Case representativeness; ③Control representativeness;④Definition of control; ⑤Comparability of study designs; ⑥Determination of exposure; ⑦The same determination method of cases and controls. A score of more than 6 is level A, otherwise level B.

#### Characteristics of included studies

The results of the literature search showed that studies investigating the association between ABO blood type and CRT exclusively focused on PICC related thrombosis. All five were retrospective cohort studies encompassing 18407 cases. Among these, 6222 individuals had O blood type, 5808 had A blood type, 4770 had B blood type, and 1607 had AB blood type. Four studies were conducted in China and one was conducted in Australia. The overall incidence of symptomatic PICC related thrombosis was determined to be 4.47% (822/18407). The primary study population comprised patients with cancer aged > 18 years old. Only Li et al. conducted regular follow-ups for CRT by using Doppler ultrasonography. In the other four studies, Doppler ultrasound was performed in patients with clinical symptoms of suspected catheter-associated thrombosis. Therefore, the study by Li et al. reported both symptomatic and asymptomatic thrombosis[10], whereas the other four studies reported only symptomatic thrombosis. Asymptomatic thrombosis is very common and has a high incidence. Combining the five studies for analysis may have missed a significant proportion of patients with asymptomatic thrombosis in the overall population with PICC-VTE. Therefore, we only combined the four studies on symptomatic thrombosis for meta-analysis and only conducted a descriptive analysis for the study by Li et al.(Table 6)

**Table 6:** The characteristics of included studies.

**Study Country Period Design CT Participant VTE type Total (C/C) Age(years) O (C/C) A (C/C) B (C/C) AB (C/C) CRT diagnostic method**													
Present study (2023)	China	Jan 2015 to Dec 2021	RCS	P	cancer patients F = 3593	SVT	5.01% (425/8477)	>18	111/2755	150/2632	112/1974	52/691	Doppler ultrasound is performed for patients with clinical symptoms of suspected catheter-associated thrombosis are present
Li et al.(2021)	China	Jan 2018 to Dec 2019	RCS	P	cancer patients F = 1299	SVT/ASVT(88/77)	7.01% (165/2353)	(53±13)/(52±14)	28/656	44/577	63/730	30/225	After PICC placement, ultrasound follow-up examination was performed at 1 week, 2 weeks, 1, 3, 6, and 12 months, respectively. If the patient has clinical symptoms related to upper extremity thrombosis, ultrasound examination should be performed at any time.
Koo et al. (2018)	Australia	Sep 2010 to Aug 2014	RCS	P	cancer patients F = 1251	SVT	4.11% (124/3020)	>18	44/1288	50/1146	22/340	8/122	Doppler ultrasound is performed for patients with clinical symptoms of suspected catheter-associated thrombosis are present
Wang-1 et al.(2019)	China	Jan 2015 to Sep 2017	RCS	P	cancer patients F = 1196	SVT	2.41% (54/2242)	>18	7/656	13/577	25/730	9/225	Doppler ultrasound is performed for patients with clinical symptoms of suspected catheter-associated thrombosis are present
Wang-2 et al.(2019)	China	Jan 2018 to Oct 2019	RCS	P	cancer patients F = 1231	SVT	5.66% (131/2315)	>18	21/656	42/577	48/726	20/225	Doppler ultrasound is performed with clinical symptoms of suspected catheter-associated thrombosis are present

RCS: retrospective cohort study; CT: catheter type; P: peripherally inserted central catheter; C/C: case/control; F: female

SVT: symptomatic venous thrombosis; ASVT: asymptomatic venous thrombosis; (mean ± standard deviation)

#### The results of meta-analysis of pairwise comparisons between different blood types for symptomatic thrombosis

**Fig 3:**
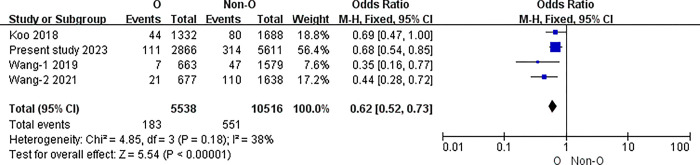
O blood type and non-O.

**Fig 4:**
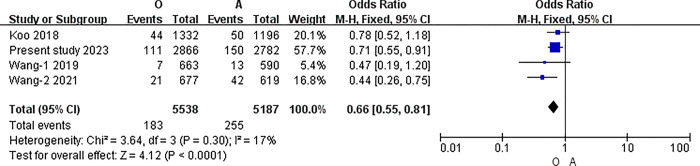
O blood type and A.

**Fig 5:**
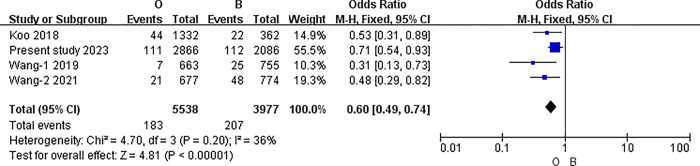
O blood type and B.

**Fig 6:**
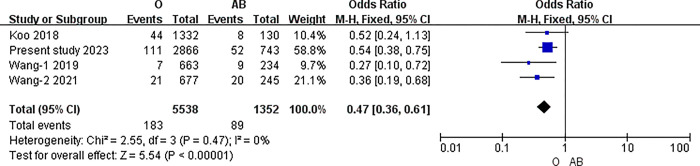
O blood type and AB.

**Fig 7:**
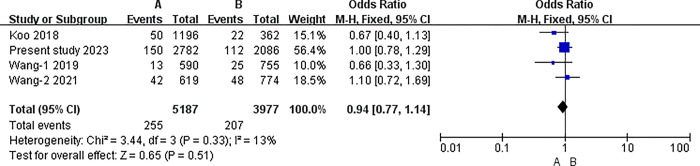
A blood type and B.

**Fig 8:**
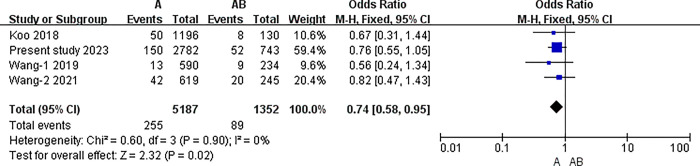
A blood type and AB.

**Fig 9:**
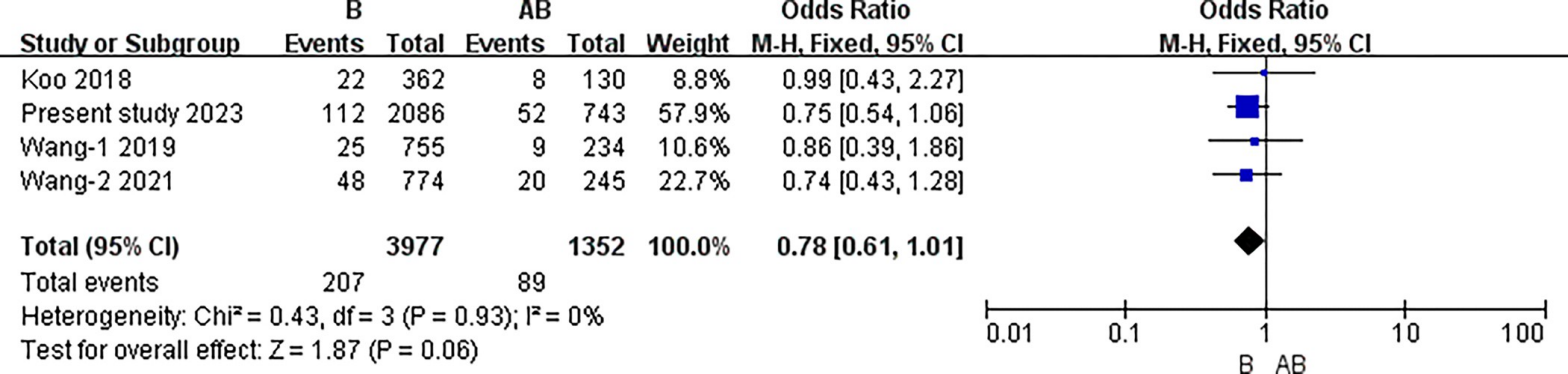
B blood type and AB.

The I2 statistic was nonsignificant in all pairwise comparisons. Hence, the fixed-effect model was used in all analyses. The results of the meta-analysis indicated that the incidence of PICC related thrombosis was significantly lower in individuals with O blood type than in those with A, B, or AB blood types (O vs. A: 3.30% vs. 4.92%, P<0.001; O vs. B: 3.30% vs. 5.20%, P<0.001; O vs. AB: 3.30% vs. 6.58%, P<0.001) (all P<0.0083). However, in the pairwise comparison between A, B, and AB, the differences were nonsignificant (A vs. B: 4.92% vs. 5.20%, P = 0.51; A vs. AB: 4.92% vs. 6.58%, P = 0.02; B vs. AB: 5.20% vs. 6.58%, P = 0.06) (all P>0.0083).(Table 7) (Figs 3–9)

**Table 7:** The results of meta-analysis for symptomatic venous thrombosis.

T1 vs. T2	Subgroup Study Total				Heterogeneity	Model	T1	T2	P value
**O vs. Non-O**		Overll	4	16054	I2 = 38%, P = 0.18	Fixed	3.30% (183/5538)	5.24%(551/10516)	<0.001
	China		3	13034	I2 = 56%, P = 0.10	Fixed	3.30%(139/4206)	5.34% (471/8828)	<0.001
	Australia		1	3020	No applicable	Fixed	3.03%(44/1332)	4.74% (80/1688)	0.05
**O vs. A**	Overll		4	10725	I2 = 17%, P = 0.30	Fixed	3.30% (183/5538)	4.92% (255/5187)	<0.001
	China		3	8197	I2 = 31%, P = 0.24	Fixed	3.30%(139/4206)	5.14%(205/3991)	<0.001
	Australia		1	2528	No applicable	Fixed	3.03%(44/1332)	4.18%(50/1196)	0.25
**O vs. B**	Overll		4	9515	I2 = 36%, P = 0.20	Fixed	3.30% (183/5538)	5.20% (207/3977)	<0.001
	China		3	7821	I2 = 55%, P = 0.11	Fixed	3.30%(139/4206)	5.12%(185/3615)	<0.001
	Australia		1	1694	No applicable	Fixed	3.03%(44/1332)	6.08%(22/362)	0.02
**O vs. AB**	Overll		4	6890	I2 = 0%, P = 0.47	Fixed	3.30% (183/5538)	6.58% (89/1352)	<0.001
	China		3	5428	I2 = 19%, P = 0.29	Fixed	3.30%(139/4206)	6.63%(81/1222)	<0.001
	Australia		1	1462	No applicable	Fixed	3.03%(44/1332)	6.15%(8/130)	0.1
**A vs. B**	Overll		4	9164	I2 = 13%, P = 0.33	Fixed	4.92% (255/5187)	5.20% (207/3977)	0.51
	China		3	7606	I2 = 0%, P = 0.44	Fixed	5.14%(205/3991)	5.12%(185/3615)	0.88
	Australia		1	1558	No applicable	Fixed	4.18%(50/1196)	6.08% (22/362)	0.13
**A vs. AB**	Overll		4	6539	I2 = 0%, P = 0.90	Fixed	4.92% (255/5187)	6.58% (89/1352)	0.02
	China		3	5213	I2 = 0%, P = 0.77	Fixed	5.14%(205/3991)	6.63%(81/1222)	0.04
	Australia		1	1326	No applicable	Fixed	4.18%(50/1196)	6.15%(8/130)	0.3
**B vs. AB**	Overll		4	5329	I2 = 0%, P = 0.93	Fixed	5.20% (207/3977)	6.58% (89/1352)	0.06
	China		3	4837	I2 = 0%, P = 0.95	Fixed	5.12%(185/3615)	6.63%(81/1222)	0.05
	Australia		1	492	No applicable	Fixed	6.08% (22/362)	6.15%(8/130)	0.98

#### Subgroup analysis for symptomatic thrombosis according to country

In China, the subgroup analysis showed that the incidence of PICC related thrombosis in individuals with O blood type was significantly lower than that in individuals with A, B, or AB blood types (all P<0.0083). However, in the pairwise comparisons among A, B and AB, the differences were nonsignificant (all P>0.0083). In Australia, no significant difference were present in pairwise comparisons among the four blood types (all P>0.0083). (Table 7)

#### Descriptive analysis for the study of Li et al

[10] The incidence of PICC related thrombosis was 7.01% (165/2353). The symptomatic and asymptomatic thrombosis was 3.74% (88/2353) and 3.27% (77/2353), respectively. The incidence of PICC related thrombosis in the O, A, B, and AB blood types was 4.09% (28/684), 7.09% (44/621), 8.53% (63/739), and 11.76% (30/255), respectively. Li et al. found that the incidence of PICC related thrombosis in blood types A (OR, 1.680; 95% CI, 1.009–2.798), B (OR, 1.835; CI, 1.137–2.961), and AB (OR, 3.275; CI, 1.840–5.829) was significantly higher than that in O blood type.

#### Funnel plot

**Fig 10:**
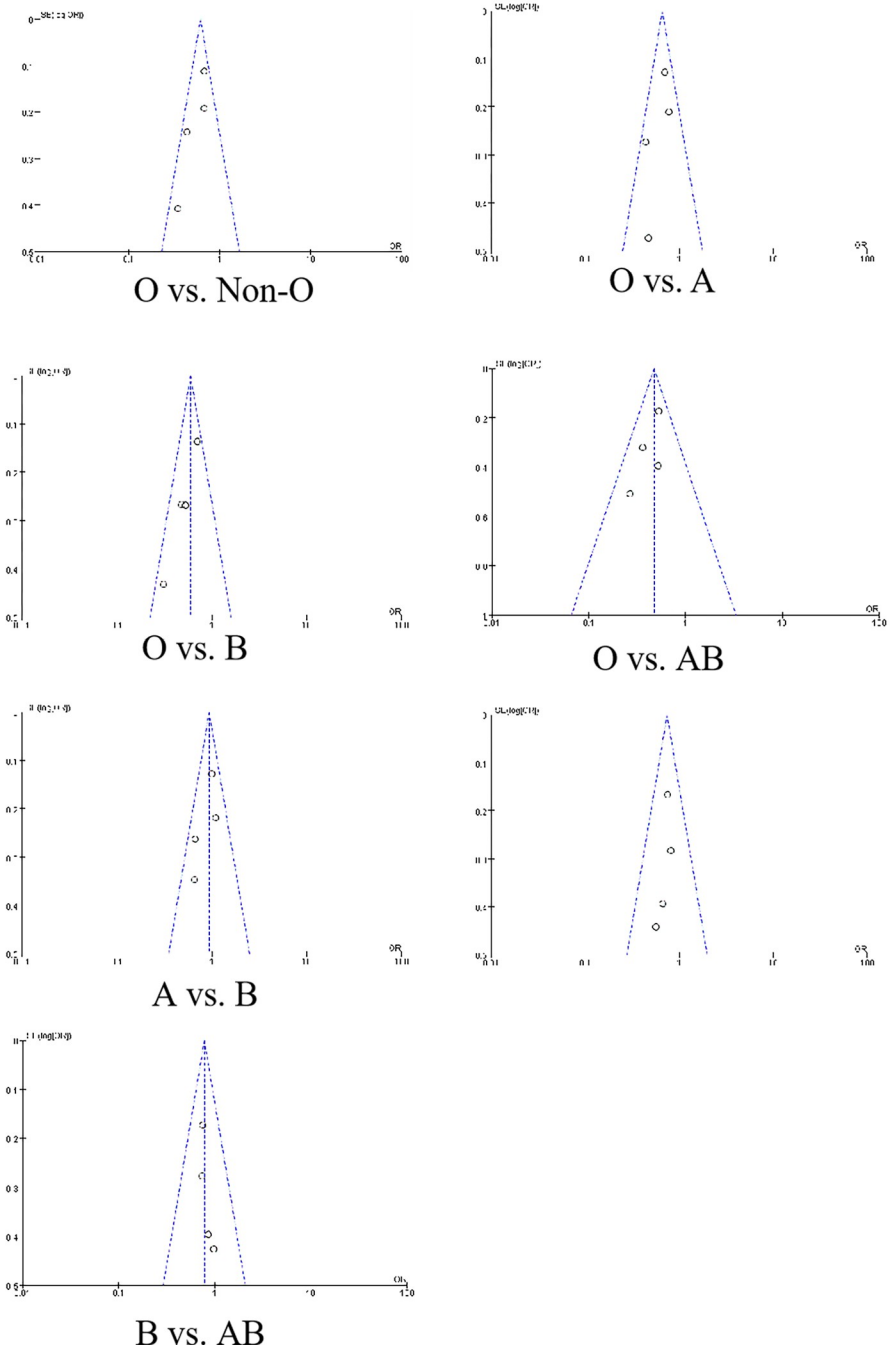
The funnel figure for publication bias.

The dots are both symmetrically and evenly distributed on both sides of X = 0 in the funnel plot. There is the possibility of an obvious small-sample effect or publication bias in this meta-analysis. (Fig 10)

## Discussion

The literature search indicated that few studies are currently available on the relationship between ABO blood type and CRT, and that these have only focused on PICC. Moreover, the results of these studies are inconsistent, and pairwise comparisons of the incidence of PICC related thrombosis among ABO blood types in existing studies has not been completed. Therefore, the relationship between the O, A, B, and AB blood types in PICC related thrombosis remains unclear. Herein, we used data from previously published studies combined with data from our hospital, a total of 18407 patients was included, which was the study with the largest data to analyze the relationship between ABO blood types and PICC related thrombosis. Hence, this review provides evidence for the significance of exploring the relationship between ABO blood type and the risk of PICC related VTE in patients with cancer.

First, we explored the risk factors for PICC related VET using a large sample from our hospital. We found that myelosuppression, radiotherapy, hematologic tumor, and non-O blood type were an independent risk factors for symptomatic PICC related thrombosis. Multiple studies have reported that radiotherapy is a high-risk factor for PICC related thrombosis[17–21]. Radiotherapy damages vascular endothelial cells, and during tumor necrosis, many coagulation factors are released, increasing the risk of thrombosis. Our study also found that patients who received radiotherapy during catheterization had a 1.52-fold increased risk of PICC-VTE compared with those who did not receive radiotherapy. Moreover, patients with myelosuppression had a 1.47-fold higher risk of PICC-VTE than patients without myelosuppression. Guan et al. also found that myelosuppression is an independent risk factor for PICC-VTE [22]. Most patients with cancer require chemotherapy, which can cause myelosuppression. Diagnosis of myelosuppression is mainly based on laboratory tests for reduced levels of white blood cells, which can increase the probability of infection and the risk of PICC-VTE. Patients with myelosuppression usually choose to stay at home, and reduced activity may increase the risk of PICC-VTE. Erythropoietin (EPO) is often administered to patients with myelosuppression, and EPO treatment increases the risk associated with CRT[23]. This may be because the rapid regeneration of blood cells increases the blood viscosity. We also found that the hematologic tumor had a 6.71-fold higher risk of PICC-VTE than breast cancer, and had a 2.59-fold higher risk than gynecological tumor. The existing study have shown that hematologic tumor was a high-risk factor for thrombosis[24]. They were more likely to have abnormalities in coagulation function and blood routine, which can further increase the risk of thrombosis[25].

The overall incidence of symptomatic PICC related thrombosis was 4.47% (822/18407). This review revealed that the incidence of symptomatic PICC related thrombosis was lower in patients with O blood type than that with non-O blood type [3.30% (183/5538) vs. 5.24% (551/10516), P<0.001]. Further pairwise comparison analysis between different ABO blood types revealed that the incidence of symptomatic PICC related thrombosis in patients with the O blood type was significantly lower than that in patients with the A, B, and AB blood types (all P<0.0083). Currently, the close correlation between ABO blood type and plasma vWF levels is considered as a possible reason for this[26,27]. The ABO blood type can influence vWF levels and their clearance from the blood. vWF is synthesized and secreted by endothelial cells and megakaryocytes, and mediates platelet adhesion and aggregation, thereby promoting thrombosis formation[28]. Patients with cancer have higher vWF levels[29], and these can vary among different ABO blood types. The O blood type had vWF levels approximately 25% lower than those in the non-O blood types[27–29]. Among the non-O blood types, the AB blood type had the highest vWF levels, followed by B and A[30–32]. In this review, the incidence of symptomatic PICC related thrombosis in patients was ranked from low to high as follows: O (3.30%), A (4.92%), B (5.20%), and AB (6.58%), which corresponded to the synergistic relationship between vWF levels among different ABO blood types. The molecular mechanism between ABO blood type and vWF is not fully understood and needs further study but is probably mediated by ABO (H) carbohydrate structure carried on the N- and O-linked glycans of vWF[33].

Additionally, the incidence of symptomatic PICC related thrombosis in patients was ranked from low to high as follows: O, A, B, and AB, which may also be related to VIII levels among ABO blood types. Studies have found significant differences in factor VIII levels among ABO blood types in the order AB>B>A>O[34,35], which corresponds to the synergistic relationship between vWF levels among different ABO blood types. This may be related to the combination of factor VIII with vWF to form the vWF-VIII factor complex, which increases the half-life and stability of factor VIII and protects this from degradation and clearance[36].

Moreover, previous studies have demonstrated that patients with non-O blood types have a higher risk of pulmonary embolism(PE) and deep venous thrombosis (DVT) [37,38], whereas those with the O blood type have an increased risk of bleeding[28]. Another study found that the O blood type had a higher APTT level than the non-O blood type[39]. To explore the relationship between ABO blood type and APTT levels, we compared APTT levels among the different blood types of 8477 patients in this study. We found that the APTT level in O blood type was higher than that in the non-O blood types [O(29.10±6.88) vs. A(27.77±6.5) vs. B(27.75±7.11) vs. AB(27.52±6.69), P<0.001]. However, the difference in APTT levels among the non-O blood types did not significantly differ. Chen et al. have also found the same conclusion[40]. Although a relationship may be present between the ABO blood type and APTT, our study found that APTT did not affect the occurrence of PICC-VTE, which was similar to the results of Cardoso et al.[41]. The molecular mechanism between ABO blood type and APTT was not fully understood and needed further study. It was now widely believed that it might be related to the levels of factor VIII in four ABO blood types. The patients with blood type O had a lower levels of factor VIII than in those with non-O blood types[42,43]. Study reported that increased factor VIII can shorten APTT[44].

We performed a subgroup analysis among different countries. Our study found that the incidence of PICC related thrombosis was higher in China than in Australia [4.68% (610/13034) vs. 4.11% (124/3020)]. In subgroup analysis, the incidence of PICC related thrombosis of O blood type was lower than that in non-O blood types in Chinese subgroup, and the difference was statistically significant (P<0.001). However, there was no significant difference in the incidence of PICC related thrombosis between O and non-O blood types in the Australian subgroup (P = 0.05). This may be related to the distribution of ABO blood types in different countries. In China, the majority of the population consists of Asians, while in Australia, the majority of the population consists of people of Caucasians. We found that in the Chinese subgroup, the proportion of non-O blood types was higher than in the Australian subgroup [67.73% (8828/13034) vs. 55.89% (1688/3020)]. And other studies have also found the same conclusion. A large-sample study of 200000 found that the proportion of non-O blood types accounted for 70.10% of the Chinese population[45]. Another study found that non-O blood types accounted for 50%-60% in Caucasians[46,47].

Li et al.[10] reported both symptomatic and asymptomatic thrombosis, where the incidence of thrombosis in O, A, B, and AB blood types was 4.09% (28/684), 7.09% (44/621), 8.53% (63/739), and 11.76% (30/255), respectively, which was higher than the incidence of symptomatic thrombosis in our meta-analysis (O: 3.30%; A:4.92%; B:5.20%; and AB:6.58%). Combining the five studies for analysis may lead to missing a significant proportion of patients who had asymptomatic thrombosis in the overall population with PICC-VTE. Therefore, we only combined the four studies on symptomatic thrombosis for meta-analysis and only conducted a descriptive analysis for the study by Li et al. The results of our meta-analysis are similar to those reported by Li et al.

Haddad et al.[13] reported a high incidence of PICC related thrombosis with 61.6% (140/227). Their study findings did not show a significant association between ABO blood type and PICC related thrombosis, which could have been influenced by the small sample size. This study population comprised only 227 patients who underwent Doppler ultrasound for suspected PICC related thrombosis, which overlooked a large proportion of patients with PICC, significantly increasing the incidence of PICC related thrombosis, and may lead to potential limitations in terms of case selection and control representation. Therefore, this study was excluded from the review.

### Limitations

This study had several limitations. Firstly, only five retrospective studies were included. Therefore, future multicenter prospective cohort studies with large sample sizes to compare the differences in PICC related thrombosis among the four ABO blood types should be conducted to provide more high-quality evidence for exploring the relationship between ABO blood type and PICC related thrombosis. Moreover, because the current research mainly aimed at symptomatic PICC related thrombosis, this meta-analysis mainly explored the relationship between symptomatic PICC related thrombosis and ABO blood type. However, most patients with PICC related thrombosis are asymptomatic. Therefore, the relationship between the ABO blood type and PICC related thrombosis (including asymptomatic thrombosis) remains unclear and requires further exploration. Furthermore, the catheter-to-vein ratio, triglyceride levels, and cancer-specific factors (cancer type, presence of hematologic spread, metastatic disease, and current treatments) may cause increased PICC related thrombosis risk. However, owing to limitations in the reported indicators of the included studies, a subgroup analysis of these factors was not performed in this review. Therefore, the influence of potential confounding factors on the results of this review cannot be excluded. This review aimed to analyze the relationship between the ABO blood type and all types of CVC-related thrombosis. However, the retrieved literature included only PICC. Hence, the relationship between the ABO blood type and all types of CVC-related thromboses could not be analyzed. Finally, most participants in this review were over 18 years of age. Hence, the relationship between the ABO blood type and CRT in children remains unclear and requires further investigation. Considering these limitations, the results of this meta-analysis should be interpreted with caution in clinical practice.

## Conclusions

According to the results from our single center analysis, we found that myelosuppression, radiotherapy, hematologic tumor, and non-O blood type were independent risk factors for symptomatic PICC related thrombosis. In the meta-analysis of further exploration of ABO blood types and PICC related thrombosis, we found that ABO blood type may indeed influence PICC related thrombosis, and the individuals with the O blood type had a lower risk of PICC related thrombosis than those with non-O blood type. However, these included studies were retrospective studies in this review, and the main outcome indicator was symptomatic thrombosis. Hence, future prospective studies are needed to explore the relationship between the ABO blood type and PICC related thrombosis.

## Supporting information

S1 Checklist (DOCX)

S1 Data (XLSX)
